# Association between the TAPSE/PASP ratio and exercise capacity in heart transplant candidates with advanced heart failure

**DOI:** 10.3389/fcvm.2025.1686578

**Published:** 2026-01-21

**Authors:** Rezzan Deniz Acar, Murat Karacam, Seda Tanyeri, Azmican Kaya, Barkin Kultursay, Deniz Mutlu, Suleyman Cagan Efe, Gulumser Sevgin Halil, Ozgur Yasar Akbal, Cem Dogan, Mehmet Kaan Kirali

**Affiliations:** 1 Department of Cardiology, Kartal Kosuyolu Research and Education Hospital, Istanbul, Türkiye; 2 Department of Cardiology, Bitlis State Hospital, Bitlis, Türkiye; 3 Department of Cardiology, Tunceli State Hospital, Tunceli, Türkiye; 4 Center for Coronary Artery Disease, Minneapolis Heart Institute and Minneapolis Heart Institute Foundation, Minneapolis, MN, United States; 5 Department of Cardiovascular Surgery, Kartal Kosuyolu Research and Education Hospital, İstanbul, Türkiye

**Keywords:** right ventricular–pulmonary arterial coupling, TAPSE/PASP ratio, heart transplantation (HTx), exercise capacity, CPET, cardiopulmonary exercise testing

## Abstract

**Background:**

Peak oxygen consumption (VO₂) is a key determinant of heart transplant eligibility in advanced heart failure (HF), reflecting integrated cardiopulmonary performance and long-term prognosis. We aimed to evaluate the association between the tricuspid annular plane systolic excursion to pulmonary artery systolic pressure (TAPSE/PASP) ratio—a non-invasive marker of right ventricular–pulmonary arterial (RV–PA) coupling—and exercise capacity, as measured by peak VO₂, in heart transplant candidates.

**Methods:**

We retrospectively analyzed 384 consecutive patients with advanced HF listed for heart transplantation between 2021 and 2023. All underwent transthoracic echocardiography, cardiopulmonary exercise testing (CPET), and right heart catheterization (RHC). Patients with LVEF >25%, severe pulmonary disease, or contraindications to CPET/RHC were excluded. Participants were stratified into tertiles by TAPSE/PASP ratio. A directed acyclic graph (DAG) guided confounder selection for multivariable linear regression assessing the association between TAPSE/PASP and peak VO₂. Cox proportional hazards models evaluated the relationship between TAPSE/PASP and a composite endpoint of death, left ventricular assist device implantation, or transplantation.

**Results:**

The mean age of the patients was 50 ± 11 years; 14% were female. Higher TAPSE/PASP tertiles were associated with longer exercise duration, higher peak VO₂, and better ventilatory efficiency. In adjusted regression analysis, TAPSE/PASP was strongly associated with peak VO₂ (effect size: 6.7; 95% CI: 5.1–8.4; *p* < 0.001). Over a median follow-up of 865 days, higher TAPSE/PASP was independently associated with lower event rates, with an adjusted hazard ratio of 0.78 (95% CI: 0.68–0.90; *p* < 0.001) per 0.1-unit increase in TAPSE/PASP.

**Conclusion:**

TAPSE/PASP, beyond its role as a surrogate of RV function, is strongly associated with exercise capacity and, in secondary analyses, with long-term outcomes in advanced HF. Incorporating TAPSE/PASP into transplant evaluation protocols may enhance risk stratification and help identify patients who require closer monitoring and tailored management.

## Graphical Abstract

**Figure:**
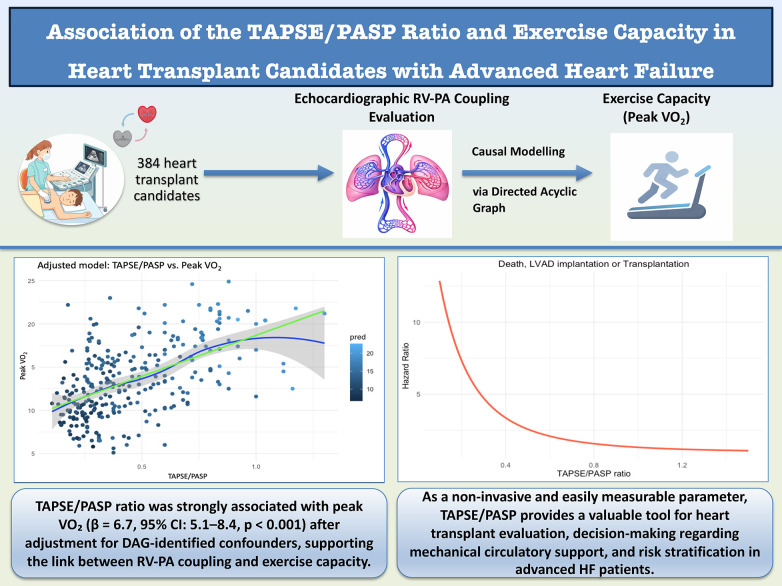
Overview of the association between the TAPSE/PASP ratio and exercise capacity in heart transplant candidates with advanced heart failure. The graphical abstract illustrates the study population, echocardiographic assessment of right ventricular–pulmonary arterial (RV–PA) coupling, causal modeling using a directed acyclic graph, and the relationship between TAPSE/PASP and peak VO_2_, as well as its association with clinical outcomes including death, LVAD implantation, or heart transplantation.

## Introduction

Heart transplantation is an established therapeutic option for selected patients with advanced heart failure (HF) who remain symptomatic despite optimal medical and device-based therapies (1). Given the persistent scarcity of donor organs, transplant candidate selection requires careful prioritization based on a comprehensive evaluation of functional status, hemodynamic stability, and overall risk profile (2). Among these parameters, exercise capacity—particularly peak oxygen consumption (VO₂)—is regarded as an important criterion in assessing transplant eligibility, as it has been reported to be associated with overall cardiopulmonary efficiency, systemic oxygen delivery, and long-term outcomes (3).

Historically, the evaluation of HF has focused primarily on left ventricular (LV) function. However, recent studies suggest that right ventricular (RV) dysfunction and RV–pulmonary artery (PA) uncoupling may be associated with disease progression, exercise intolerance, and adverse outcomes (4, 5). Among non-invasive echocardiographic parameters, the tricuspid annular plane systolic excursion to pulmonary artery systolic pressure (TAPSE/PASP) ratio was first validated by Tello et al. as a surrogate marker of RV–PA coupling (6), building upon earlier work demonstrating its correlation with hemodynamic status, functional limitation, and clinical outcomes (7).

A reduced TAPSE/PASP ratio may reflect a combination of impaired stroke volume augmentation, increased pulmonary vascular resistance, and elevated RV afterload—pathophysiological mechanisms that can limit cardiac output during exercise and contribute to ventilatory inefficiency (8, 9). While the prognostic value of TAPSE/PASP in HF populations has been increasingly recognized, its direct association with exercise capacity in heart transplant candidates has not been thoroughly investigated (10).

The present study aimed to examine the relationship between the TAPSE/PASP ratio and peak VO₂ in heart transplant candidates with advanced HF, using a directed acyclic graph (DAG) approach to optimize confounder selection and improve confounder control. In addition, we assessed the prognostic relevance of TAPSE/PASP for predicting a composite endpoint of death, left ventricular assist device (LVAD) implantation, or heart transplantation, and its potential role in transplant evaluation strategies.

## Materials and methods

### Study population

A total of 384 patients admitted to the heart transplantation outpatient clinic between 2021 and 2023, who underwent comprehensive echocardiographic examination, CPET, and RHC, were included in this study. Patients with an LVEF >25%, severe lung disease, or those with contraindications to CPET or RHC were excluded from the study. To ensure an accurate assessment of exercise capacity, patients with a peak RER below 1.05, indicating submaximal test, were excluded.

All patients fulfilled the criteria for advanced heart failure in accordance with contemporary ISHLT 2024 and ESC 2021 recommendations (2, 11). In particular, all included individuals met at least two major indicators of advanced HF, including persistent New York Heart Association (NYHA) class III–IV symptoms despite maximally tolerated guideline-directed medical therapy (GDMT), left ventricular ejection fraction ≤25%, recurrent HF hospitalizations within the preceding 12 months, peak VO₂ < 14 mL/kg/min on cardiopulmonary exercise testing, or hemodynamic compromise on right heart catheterization (elevated filling pressures and/or cardiac index <2.2 L/min/m^2^). All patients were receiving GDMT according to ESC and ISHLT recommendations, including beta-blockers, Angiotensin-converting enzyme inhibitor (ACEI)/Angiotensin receptor blocker (ARB)/Angiotensin receptor–neprilysin inhibitor (ARNI), mineralocorticoid receptor antagonists, and SGLT2 inhibitors, unless contraindicated or not tolerated. Device therapies [implantable cardioverter-defibrillator (ICD) and cardiac resynchronization therapy (CRT)] were present in patients who met guideline criteria for implantation. These treatment patterns reflect standard practice in our center for patients with advanced HF undergoing transplant evaluation.

Clinical, laboratory, echocardiographic, and catheterization data were obtained within a maximum of 2 weeks from the CPET date. The primary outcome of the study was peak VO₂, assessed via CPET. The secondary outcome was a composite of all-cause mortality, LVAD implantation, or heart transplantation during follow-up. Demographics and laboratory results were retrieved from electronic hospital records. Since the primary aim was pathophysiological understanding rather than developing a purely predictive model, missing data were not imputed, and patients with missing values were excluded. All exclusion criteria were already applied before constructing the final cohort. As CPET, echocardiographic, hemodynamic, and laboratory assessments were obtained within a standardized pre-transplant protocol, the extent of missing data was minimal. This process resulted in a complete-case cohort of 384 patients. Local ethics committee approval was obtained and the study complied with the Declaration of Helsinki. A STROBE-compliant flow diagram summarizing patient screening, exclusions, and inclusion in the final cohort is presented in Supplementary Figure S1.

### Echocardiography

Left ventricle ejection fraction (LVEF) was measured using the biplane method of disk summation (modified Simpson's rule). Doppler echocardiography was performed in accordance with current guidelines by a single experienced cardiology echocardiographer, using the EPIQ CVx v9.0.5 system and X5-1 transducer (Philips Medical Systems, Andover, MA, USA). Tricuspid annular plane systolic excursion (TAPSE) was measured using M-mode imaging in the apical four-chamber view, focusing on the right ventricle. The echocardiographic estimation of pulmonary artery systolic pressure (PASP) was determined by summing the peak velocity of tricuspid regurgitation (calculated using the Bernoulli equation) and the estimated central venous pressure, which was derived from the inferior vena cava (IVC) diameter and its collapsibility. All measurements were performed according to the American Society of Echocardiography guidelines (12, 13).

### Exercise testing

A maximal exercise test was performed, and the exercise capacity was expressed in (metabolic equivalent) METs, with the maximal effort provided by the patient. The maximal cardiopulmonary exercise test was conducted using a continuous, incremental treadmill protocol based on an individualized ramp design, on a JAEGER Vyntus CPX system (Vyaire Medical, Germany). Oxygen uptake was measured breath by breath using an automated system, with data collected at rest, during graded exercise, and throughout a 2-min recovery period. METs were calculated by dividing the VO_₂_ max value by 3.5 mL/kg/min. VO₂, VCO₂, and the RER (VCO₂/VO_₂_, RER) were computed and averaged every 10 s. Maximal effort on the treadmill test was specified as an RER of greater than 1.05. Peak VO_₂_ was defined as the highest 10-s averaged VO_₂_ during the last stage of the exercise test. Blood pressure was measured periodically before and throughout the test every 3 min and during the recovery phase.

### Cardiac catheterization

Right heart catheterization was performed under stable conditions, without inotropes or acute vasodilators. Vasoreactivity testing was not routinely performed. All patients were on optimized guideline-directed medical therapy at the time of invasive assessment. A 7Fr balloon-tipped Swan–Ganz catheter (Edwards Lifesciences, Irvine, CA, USA) or pigtail catheter was used through the right jugular or femoral vein for RHC. The indirect Fick method was used for cardiac output estimation. All pressure tracings were visually inspected for physiological accuracy, and end-expiratory pressure values were recorded.

### Statistical analyses

Normally distributed continuous data were expressed as mean and SD values, whereas non-normally distributed data were expressed as medians and interquartile ranges. Categorical data were described as absolute and percentage values. Normality of the data was determined using histograms and the Shapiro–Wilk test. TAPSE/PASP tertiles were defined based on the distribution of values in the cohort: Tertile 1 (≤0.32), Tertile 2 (0.33–0.55), and Tertile 3 (≥0.56). Baseline characteristics and clinical, echocardiographic, and catheterization variables were compared across tertiles. With regard to sample distribution, analysis of variance and Kruskal–Wallis tests were used for comparison of continuous data groups according to TAPSE/PASP tertiles, and Pearson *χ*^2^ or Fisher's exact test was used for comparison of categorical data groups. Group comparisons were performed using *post-hoc* tests of Tukey and Dwass–Steel–Critchlow–Fligner (DSCF), as appropriate.

The continuous association between TAPSE/PASP and peak VO₂ was assessed using multivariable linear regression. For analysis with TAPSE/PASP as the primary predictor variable, linear regression was adjusted for demographics and co-morbidities, with covariates selected based on *a priori* knowledge (14–18). A DAG was used to identify assumed relationships between variables and detect potential confounders (19) (Supplementary Figure S2).

The DAG was developed using established physiological knowledge and expert consensus. Variables identified as potential confounders included age, sex, body mass index (BMI), hemoglobin, ischemic etiology, pulmonary vascular resistance (PVR), and cardiac index (CI), owing to their known associations with both right ventricular function and systemic oxygen delivery. ProBNP was considered a potential mediator. To avoid collider bias, variables influenced by both TAPSE/PASP and peak VO_₂_ (e.g., exercise-induced pulmonary pressures) were excluded from the model.

Based on the DAG, multivariable linear regression was performed to estimate the direct and indirect associations between TAPSE/PASP and peak VO_₂_. The association between TAPSE/PASP and the composite endpoint (death, LVAD implantation, or transplantation) was evaluated using multivariable Cox models. Covariates were selected on the basis of clinical plausibility and prior evidence in advanced HF. Peak VO_₂_ was not included in the Cox models, as it was considered a potential mediator in the pathway between TAPSE/PASP and clinical outcomes. Results are reported as hazard ratios (HR) with 95% confidence intervals (CI).

All analyses were performed using a complete-case approach. After applying predefined exclusion criteria, all variables included in the regression and Cox models were complete, and no imputation was required. Patients with missing values in any of the variables required for the analytic models were excluded during cohort construction; therefore, no missing data were present in the final dataset.

All statistical analyses were conducted in RStudio version 4.3.1 (R Foundation for Statistical Computing, Vienna, Austria) using the packages “rms,” “effects,” “daggity,” “ggplot2,” “survival,” and “survminer.”

## Results

Among the 384 patients included in this analysis, the mean age was 50 ± 11 years, with a small proportion being female (14%). The mean value of LVEF was 22 ± 6.2%, and TAPSE was 1.7 ± 0.4 cm. The median TAPSE/PASP values are 0.23, 0.40, and 0.77 for T1, T2, and T3, respectively.

Baseline demographic and echocardiographic variables are shown in Table 1 according to TAPSE/PASP tertiles. A significant difference in BMI was observed across tertiles, with the highest BMI seen in T3 (*p* = 0.007). The ischemic etiology of heart failure increased across tertiles, with 44.4% of patients in T1, 61.6% in T2, and 65.6% in T3 having ischemic heart disease (*p* = 0.002). The mean LVEF significantly differed across tertiles. T1 had a mean LVEF of 20.9 ± 3.62%, T2 had 21.6 ± 4.01%, and T3 had 23.3 ± 5.22% (*p* < 0.001). The LVEDD and LVESD did not differ across tertiles. The severity of both mitral regurgitation (MR) and tricuspid regurgitation (TR) decreased across tertiles, with milder grades more prevalent in higher tertiles. IVC diameter and plethora showed a decreasing trend across tertiles, with significantly lower values in the higher tertiles (*p* < 0.001).

**Table 1:** Demographic and echocardiographic data of patients by tertiles.

Variable	T1 (≤0.32, *n* = 126)	T2 (0.33–0.55, *n* = 129)	T3 (≥0.56, *n* = 129)	*p*-Value
Gender, male (%)	107 (84.9%)	113 (87.6)	107 (82.9)	0.573
Age, years	51.9 (10.5)	50.3 (11.3)	49.8 (11.6)	0.270
BMI, kg/m^2^	26.6 (4.5)	27.4 (4.85)	28.5 (5.39)	0.007^b^
Smoker, *n* (%)	25 (20.5)	38 (31.4)	33 (30)	0.117
Ischemic etiology, *n* (%)	56 (44.4)	77 (61.6)	80 (65.6)	0.002
DM, *n* (%)	52 (44.1)	38 (32.5)	40 (33.6)	0.126
Ejection fraction, (%)	20.9 (3.62)	21.6 (4.01)	23.3 (5.22)	<0.001^b^^,^^c^
LVEDD, cm	6.82 (0.81)	6.88 (0.81)	6.79 (1.04)	0.750
LVESD, cm	6.06 (0.88)	6.03 (0.86)	5.84 (1.09)	0.128
LA, cm	4.75 (0.45)	4.71 (0.57)	4.36 (0.65)	<0.001^b^^,^^c^
MR grading				<0.001
0	1 (0.8)	0 (0)	0 (0)	
1	20 (16.5)	43 (34.4)	76 (59.4)	
2	63 (52.1)	52 (41.6)	46 (35.9)	
3	37 (30.6)	30 (24)	6 (4.7)	
TR grading				<0.001
1	28 (22.2)	63 (48.8)	127 (98.4)	
2	64 (50.8)	50 (38.8)	2 (1.6)	
3	34 (27)	16 (12.4)	0 (0)	
LVDD grading				<0.001
0	1 (0.8)	0 (0)	0 (0)	
1	6 (5)	11 (9.2)	68 (54)	
2	18 (14.9)	29 (24.2)	32 (25.4)	
3	96 (79.3)	80 (66.7)	26 (20.6)	
TAPSE, cm	1.35 (0.3)	1.66 (0.33)	2.12 (0.38)	<0.001^a^^,^^b^^,^^c^
PASP, mm Hg	58.9 (12)	42 (9.22)	28 (5.17)	<0.001^a^^,^^b^^,^^c^
TAPSE/PASP, mm/mmHg	0.23 (0.04)	0.40 (0.06)	0.77 (0.1)	<0.001^a^^,^^b^^,^^c^
IVC, cm	2.21 (0.48)	1.95 (0.4)	1.6 (0.31)	<0.001^a^^,^^b^^,^^c^
Plethorea, *n* (%)	65 (53.7)	28 (23.5)	2 (1.7)	<0.001
LVAD, *n* (%)	29 (23)	19 (14.7)	6 (4.7)	<0.001
Heart Tx, *n* (%)	3 (2.4)	0 (0)	0 (0)	0.035
Mortality	49 (38.9)	42 (32.5)	35 (27.7)	<0.001

BMI, body mass index; DM, diabetes mellitus; IVC, inferior vena cava; LA, left atrium; LVDD, left ventricular diastolic dysfunction; LVEDD, left ventricular end-diastolic diameter; LVESD, left ventricular end systolic diameter; MR, mitral regurgitation; PASP, pulmonary arterial systolic pressure; TAPSE, tricuspid annular plane systolic excursion; TR, tricuspid regurgitation.

a T1 vs. T2.

b T1 vs. T3.

c T2 vs. T3.

In terms of pharmacological therapy, ACEI/ARB were used in 114 patients (29.7%), ARNI in 97 (25.3%), beta-blockers in 357 (93.0%), MRAs in 311 (81.0%), SGLT2 inhibitors in 180 (46.9%), and loop diuretics in 365 (95.1%). Anticoagulation therapy was prescribed in 142 patients (37.0%) and statins in 162 (42.2%). Device therapy was also prevalent: ICD implantation was present in 189 patients (49.2%) and CRT in 40 patients (10.4%). These treatment patterns are consistent with contemporary guideline-directed management of advanced HF and reflect standard practice in our transplant evaluation program (Supplementary Table S1**)**.

As tertiles increased, left ventricular end-diastolic pressure, pulmonary artery systolic and mean pressures, right atrial pressure, trans-pulmonary gradient, and pulmonary vascular resistance (PVR) showed a decreasing trend, while trans-systemic gradient, stroke volume (SV), stroke volume index (SVI), cardiac output (CO), and CI progressively increased (*p* < 0.05 for all) (Table 2). In a supplementary correlation analysis, echocardiographic PASP demonstrated a strong, significant correlation with invasively measured PASP (*r* = 0.615, *p* < 0.001). Likewise, echocardiographic TAPSE/PASP showed high concordance with invasively derived TAPSE/PASP (*r* = 0.766, *p* < 0.001). These findings support the reliability of non-invasive RV–PA coupling assessment and validate the use of echocardiographic measurements in this cohort (Supplementary Figure S3).

**Table 2:** Cardiac catheterization data of patients by tertiles.

Variable	T1 (*n* = 126)	T2 (*n* = 129)	T3 (*n* = 129)	*p*-Value
LVEDP, mm Hg	26.2 (6.69)	24.3 (8.75)	17.2 (8.21)	<0.001^b^^,^^c^
PASP, mm Hg	63.4 (16.6)	48.9 (14.6)	37.9 (15.1)	<0.001^a^^,^^b^^,^^c^
PAMP, mm Hg	41.2 (10.5)	32.7 (10.7)	23.4 (10.7)	<0.001^a^^,^^b^^,^^c^
RAP, mm Hg	12.6 (6.37)	10.4 (5.56)	6.14 (3.09)	<0.001^a^^,^^b^^,^^c^
TPG, mm Hg	15.1 (8.45)	8.59 (4.93)	6.32 (4.89)	<0.001^a^^,^^b^^,^^c^
TSG, mm Hg	73.7 (17.6)	74.1 (14.1)	86 (15)	<0.001^b^^,^^c^
SV, mL/beat	38.5 (11.5)	40.4 (12.9)	52.3 (15.6)	<0.001^b^^,^^c^
SVI, mL/m^2^/beat	20.2 (6.22)	20.6 (6.02)	26.8 (8.25)	0.001^b^^,^^c^
CO, L/min	3.20 (0.88)	3.37 (0.95)	4.06 (1.13)	<0.001^b^^,^^c^
CI, L/min/m^2^	1.66 (0.42)	1.72 (0.42)	2.05 (0.49)	<0.001^b^^,^^c^
PVR, Woods unit	5.04 (3.2)	2.79 (1.82)	1.67 (1.54)	<0.001^a^^,^^b^^,^^c^
SVR, Woods unit	24.5 (6.77)	23.4 (6.39)	22.3 (7.18)	0.069
RVSWI, mm Hg × mL × m^−2^	8.21 (3.95)	6.62 (2.7)	6.46 (2.9)	<0.001^a^^,^^b^

CI, cardiac index; CO, cardiac output; LVEDP, left ventricular end-diastolic pressure; PAMP, pulmonary arterial mean pressure; PASP, pulmonary arterial systolic pressure; PVR, pulmonary vascular resistance; RAP, right atrial pressure; RVSWI, right ventricular stroke work index; SV, stroke volume; SVI, stroke volume index; SVR, systemic vascular resistance; TPG, trans-pulmonary gradient; TSG, trans-systemic gradient.

a T1 vs. T2.

b T1 vs. T3.

c T2 vs. T3.

Among the laboratory parameters, glomerular filtration rate (GFR), albumin, and hemoglobin increased across tertiles, while liver enzymes and proBNP showed a decreasing trend (*p* < 0.05 for all) (Table 3).

**Table 3:** Laboratory data of patients by tertiles.

Variable	T1 (*n* = 126)	T2 (*n* = 129)	T3 (*n* = 129)	*p*-Value
Creatinine, mg/dL	1.13 (0.49)	1.04 (0.31)	1.05 (0.48)	0.211
GFR, mL/min	77.8 (22.5)	84.5 (21.7)	85 (23.4)	0.020^a^^,^^b^
ALT, U/L	29.5 (13.4–30.6)	22.6 (16.5–32.4)	17.8 (13.5–24.8)	0.022
Total bilirubin, mg/dL	1 (0.69–1.56)	0.84 (0.57–1.19)	0.51 (0.35–0.68)	<0.001^a^^,^^b^^,^^c^
GGT, U/L	54 (28.5–91.5)	38 (25.8–73.3)	24 (16.3–34)	<0.001^a^^,^^c^
ProBNP, ng/L	3,370 (2,082–5,576)	2,348 (1,048–3,969)	917 (485–1,763)	<0.001^a^^,^^b^^,^^c^
Triglyceride, mg/dL	96.4 (72.4–125)	126 (92.7–175)	147 (106–244)	<0.001^a^^,^^b^^,^^c^
LDL, mg/dL	75.9 (56.4–94.3)	101 (80.8–124)	106 (74.7–129)	<0.001^b^^,^^c^
HDL, mg/dL	35.2 (28.7–46)	39.3 (33.1–48.5)	43.5 (33.9–51.7)	<0.001^b^
Total protein, g/L	70.7 (6.75)	71 (6.17)	71.3 (9.32)	0.863
Albumin, g/L	42.6 (5.17)	43.5 (4.44)	44.8 (6.28)	0.016^b^
INR	1.31 (1.18–1.54)	1.23 (1.1–1.41)	1.08 (1.01–1.18)	<0.001^a^^,^^b^^,^^c^
Hemoglobin, g/dL	13.2 (2.37)	13.8 (2.06)	14.1 (1.61)	0.002^a^^,^^b^
Hematocrit, %	41.7 (6.69)	43.1 (5.62)	43.1 (4.78)	0.076
Platelet, 10^3^/µL	248 (205–292)	242 (202–285)	256 (221–292)	0.249

ALT, alanine aminotransferase; GFR, glomerular filtration rate; GGT, gamma-glutamyl transferase; HCT, hematocrit; HDL, high-density lipoprotein; Hgb, hemoglobin; INR, international normalized ratio; LDL, low-density lipoprotein; ProBNP, pro brain natriuretic peptide.

a T1 vs. T2.

b T1 vs. T3.

c T2 vs. T3.

**Figure 1:**
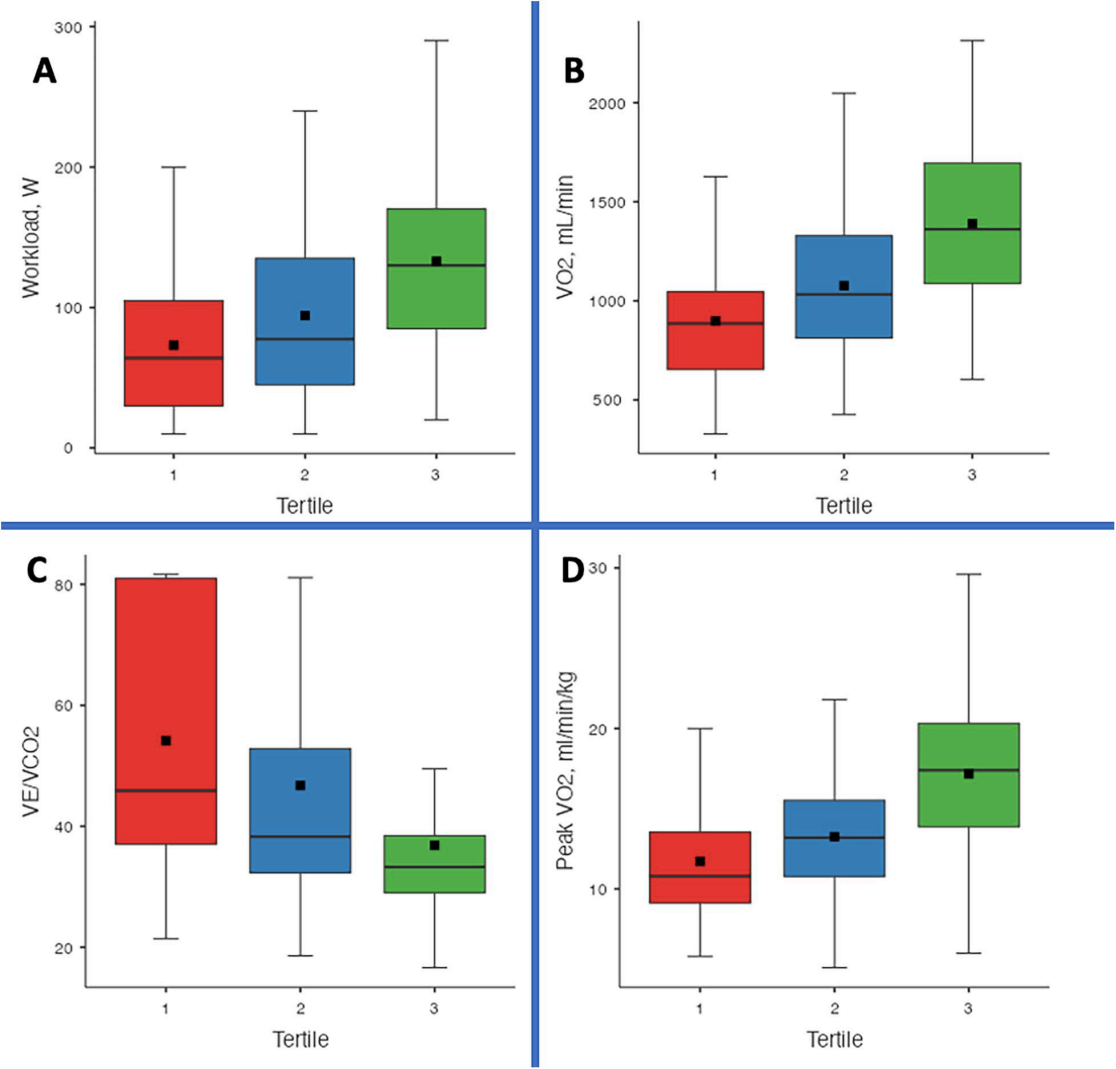
Comparison of cardiopulmonary exercise testing (CPET) parameters across TAPSE/PASP tertiles. Box-and-whisker plots depict **(A)** workload (W), **(B)** absolute oxygen consumption (VO_₂_, mL/min), **(C)** ventilatory efficiency (VE/VCO_₂_ slope), and **(D)** weight-adjusted peak oxygen consumption (peak VO_₂_, mL/min/kg) in patients with advanced heart failure awaiting heart transplantation or LVAD implantation. Horizontal lines represent medians, boxes indicate interquartile ranges, whiskers denote the 10th–90th percentiles, and black squares mark means. Higher TAPSE/PASP tertiles were generally associated with greater exercise capacity and more favorable ventilatory efficiency profiles (all *p* < 0.001).

CPET parameters showed a significant improvement across tertiles (Figure 1). Exercise duration increased from 5.39 ± 2.87 min in T1 to 6.38 ± 2.83 min in T2 and 8.49 ± 2.78 min in T3 (*p* < 0.001). Similarly, workload increased from 64 W (30–105) in T1 to 77.5 W (45–135) in T2 and 130 W (85–170) in T3 (*p* < 0.001). VE increased from 44.6 ± 12.7 L/min in T1 to 47 ± 11.1 L/min in T2 and 50 ± 12 L/min in T3 (*p* = 0.001). VO₂ improved from 898 ± 309 mL/min in T1 to 1,077 ± 368 mL/min in T2 and 1,390 ± 404 mL/min in T3 (*p* < 0.001). Peak VO₂/kg showed a similar increasing trend, rising from 11.7 ± 3.92 mL/min/kg in T1 to 13.3 ± 3.69 mL/min/kg in T2 and 17.2 ± 4.32 mL/min/kg in T3 (*p* < 0.001). METs also increased significantly from 3.35 ± 1.12 in T1 to 3.79 ± 1.06 in T2 and 4.89 ± 1.26 in T3 (*p* < 0.001) (Table 4).

**Table 4:** CPET data of patients by tertiles.

Variable	T1 (≤ 0.32, *n* = 126)	T2 (0.33–0.55, *n* = 129)	T3 (≥0.56, *n* = 129)	*p*-Value
Time, min	5.39 (2.87)	6.38 (2.83)	8.49 (2.78)	<0.001^a^^,^^b^^,^^c^
Load, watts	64 (30–105)	77.5 (45–135)	130 (85–170)	<0.001^a^^,^^b^^,^^c^
VE, L/min	44.6 (12.7)	47 (11.1)	50 (12)	0.001^b^
VO_2_, mL/min	898 (309)	1,077 (368)	1,390 (404)	<0.001^a^^,^^b^^,^^c^
Peak VO_2_, mL/min/kg	11.7 (3.92)	13.3 (3.69)	17.2 (4.32)	<0.001^a^^,^^b^^,^^c^
Pred, %	31.3 (8.65)	31.4 (10)	34.8 (16.9)	0.041^b^
RER	1.04 (0.08)	1.04 (0.07)	1.02 (0.07)	0.023^b^
METs	3.35 (1.12)	3.79 (1.06)	4.89 (1.26)	<0.001^a^^,^^b^^,^^c^
HR, 1/min	111 (24.1)	115 (28.1)	123 (23.2)	<0.001^b^^,^^c^
HRR, 1/min	59.4 (23.9)	59.5 (25)	47.6 (23.3)	<0.001^b^^,^^c^
Peak saturation, %	97 (93–99)	98 (94.8–99)	98 (96–99)	0.039^b^
VECO2 slope, VE/VCO2	54.2 (20.4)	46.8 (20.1)	36.8 (15)	<0.001^a^^,^ ^b^^,^ ^c^
VO2W slope, mL/min/W	2.7 (0.6–4.21)	3.3 (1.52–5.71)	4.32 (2.9–5.67)	0.001^b^
HRO2W slope, 1/mL/kg	1.77 (0–2.97)	2.05 (0.83–3.22)	2.55 (1.64–3.29)	0.005^b^

HR, heart rate; HRR, heart rate reserve; METs, metabolic equivalents; Pred %, percentage of predicted peak oxygen consumption; RER, respiratory exchange ratio; VE, ventilation, VE/VCO_2_, ventilatory response; VO_2_, oxygen consumption.

a T1 vs. T2.

b T1 vs. T3.

c T2 vs. T3.

The VE/VCO₂ slope significantly decreased across tertiles. The VE/VCO₂ slope was 54.2 ± 20.4 in T1, 46.8 ± 20.1 in T2, and 36.8 ± 15 in T3 (*p* < 0.001) (Table 4). To further evaluate the physiological relationship between RV–PA coupling and ventilatory efficiency, we performed an additional multivariable linear regression analysis using the same minimally sufficient adjustment set derived from the DAG. TAPSE/PASP was identified as a strong, independent determinant of the VE/VCO₂ slope (*β* = –21.19 ± 4.96; *p* < 0.001) (Supplementary Table S2).

A correlation matrix including TAPSE/PASP, peak VO₂, and VE/VCO₂ slope demonstrated the following:

- TAPSE/PASP ↔ VE/VCO₂ slope: r = –0.395, *p* < 0.001
- TAPSE/PASP ↔ peak VO₂: r = 0.524, *p* < 0.001
- VE/VCO₂ slope ↔ peak VO₂: r = –0.599, *p* < 0.001

These findings confirm that TAPSE/PASP demonstrates a stronger inverse association, indicating that RV–PA coupling influences ventilatory dynamics. These results are provided in Supplementary Table S3**.**

In an additional analysis, we evaluated whether the association between TAPSE/PASP and exercise capacity persisted when peak VO₂ was expressed as percent-predicted values. Using the same minimally sufficient adjustment set, TAPSE/PASP remained independently associated with higher % predicted peak VO₂ (*β* = 5.37 ± 2.60; *p* = 0.040), supporting the robustness of this relationship across different VO₂ scaling methods (Supplementary Table S4).

**Figure 2:**
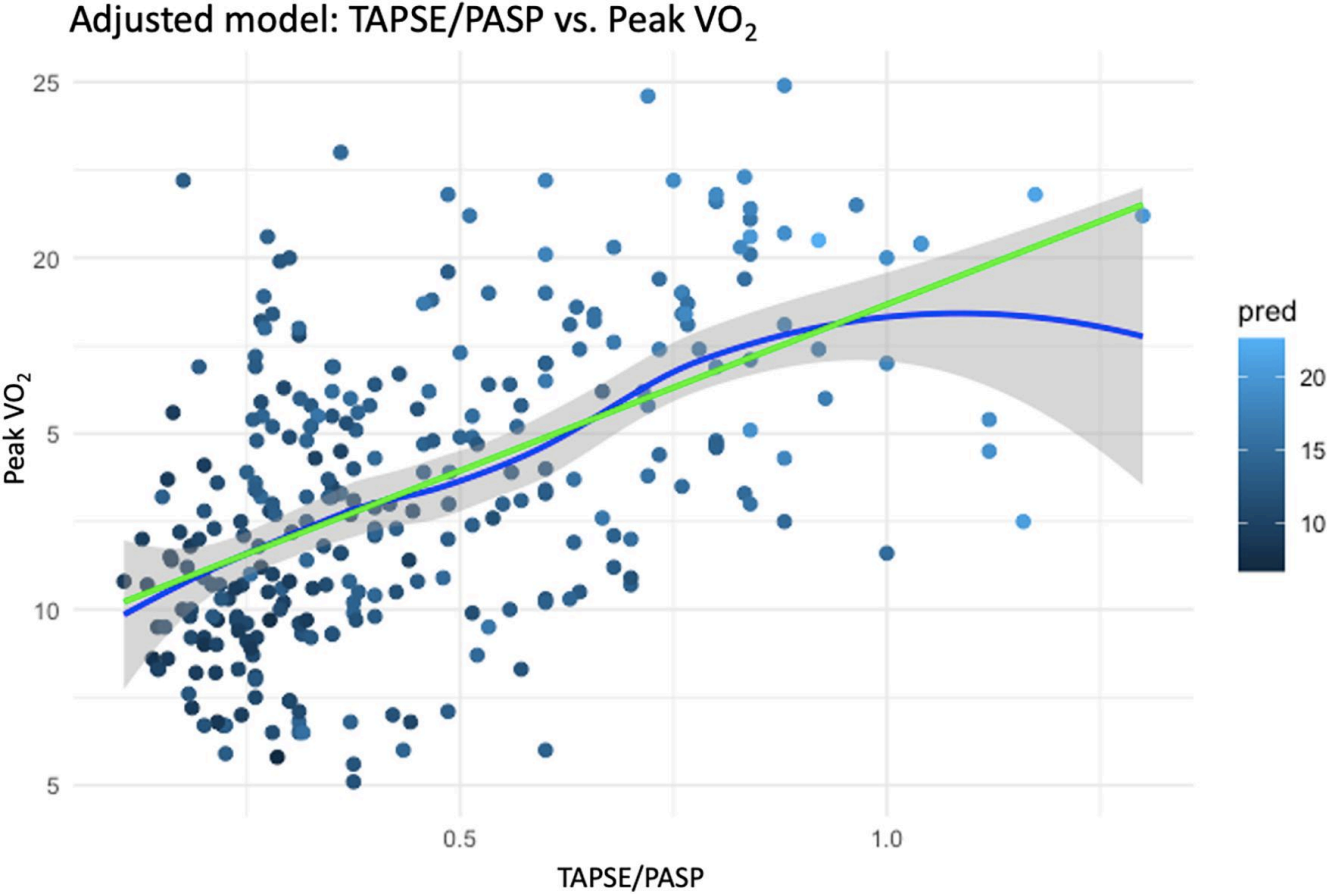
Adjusted association between TAPSE/PASP ratio and peak VO_₂_. Scatter plot with fitted regression lines showing the relationship between TAPSE/PASP and peak VO_₂_ after adjustment for confounders identified in the DAG. The green line represents the adjusted linear fit, and the blue line represents a spline-based fit with 95% confidence intervals (shaded area). A higher TAPSE/PASP ratio was strongly associated with improved peak VO_₂_ (*β* = 6.7; 95% CI, 5.1–8.4; *p* < 0.001).

In the DAG-based multiple linear regression model (Supplementary Figure S2), the TAPSE/PASP ratio was found to be associated with peak VO₂ after adjusting for confounders identified through the DAG framework, including age, gender, BMI, ischemic etiology, PVR, CI and hemoglobin levels. The estimated effect size was 6.7 (95% CI: 5.1–8.4, *p* < 0.001) (Table 5), highlighting a strong association between RV–PA coupling and exercise capacity in this cohort (Figure 2).

**Table 5:** Multiple linear regression analysis for peak VO_2._

Variable	Estimate (CI 95%)	Std. error	T value	*p*-Value
TAPSE/PASP (mm/mm Hg)	6.7 (5.1–8.4)	0.859	7.89	<.001
Gender (male)	0.64 (−0.38–1.67)	0.524	1.23	0.219
Age	−0.058 (−0.09 - −0.025)	0.016	−3.48	<.001
BMI (kg/m^2^)	−0.145 (−0.22 - −0.077)	0.034	−4.19	<.001
Ischemic etiology	0.73 (−0.02–1.48)	0.384	1.90	0.058
PVR (WU)	−0.109 (−0.26–0.04)	0.08	−1.42	0.158
Hemoglobin (g/dL)	0.49 (0.3–0.67)	0.09	5.34	<0.001
Cardiac index (L/min/m^2^)	1.627 (0.8–2.4)	0.41	3.93	<.001

BMI, body mass index; CI, confidence interval; PASP, pulmonary artery systolic pressure; PVR, pulmonary vascular resistance; TAPSE, tricuspid annular plane systolic excursion.

**Figure 3:**
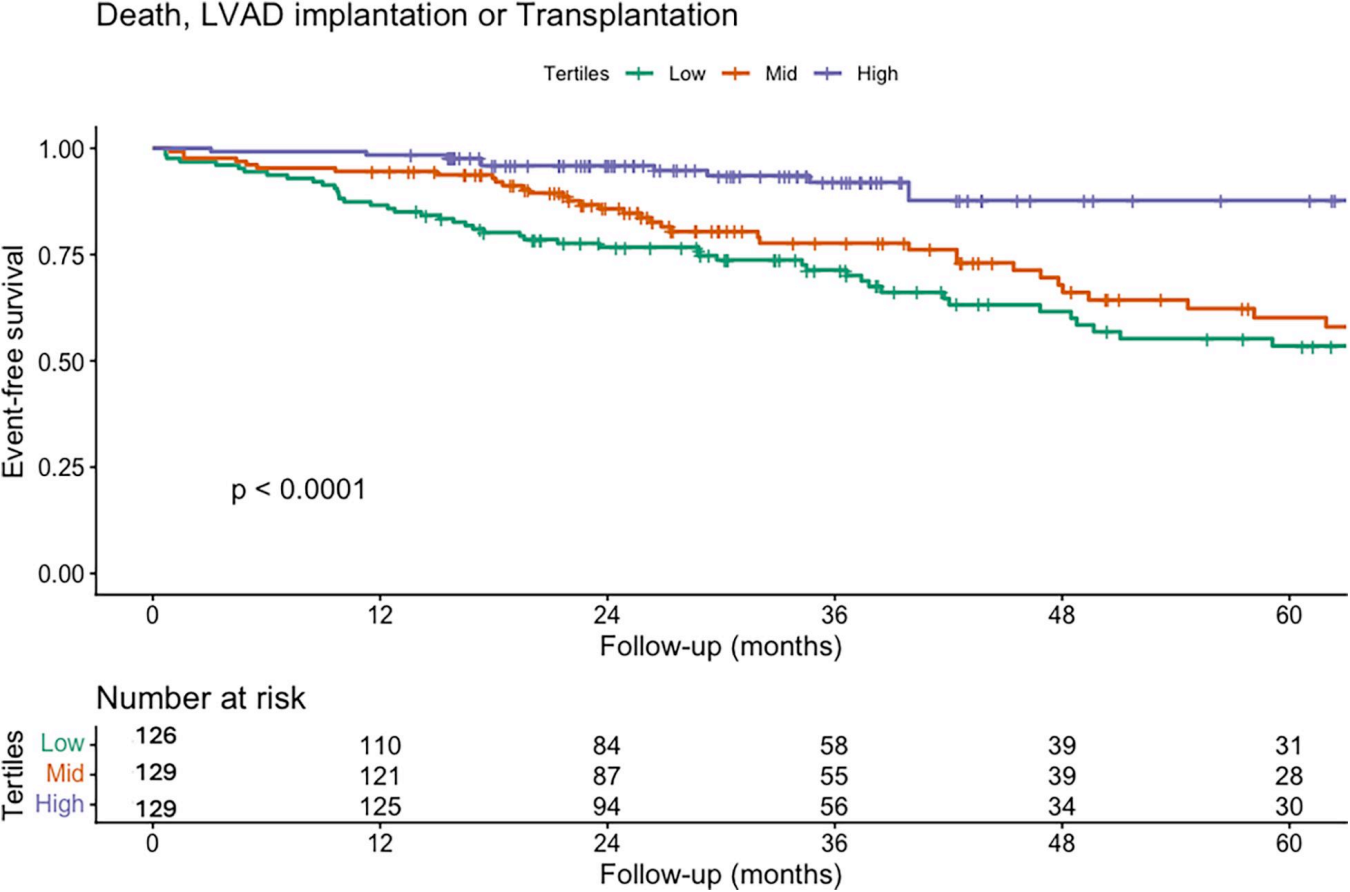
Kaplan–Meier survival curves for the composite endpoint of death, LVAD implantation, or transplantation according to TAPSE/PASP tertiles. Kaplan–Meier curves illustrate event-free survival for the composite endpoint of all-cause death, LVAD implantation, or heart transplantation, stratified by TAPSE/PASP tertiles: low tertile (green), mid tertile (red), and high tertile (purple). Patients in the higher tertile demonstrated significantly better event-free survival compared with those in the lower tertiles (log-rank *p* < 0.001). These findings indicate that impaired RV–PA coupling, as reflected by a lower TAPSE/PASP ratio, is associated with a higher incidence of adverse outcomes.

As a secondary exploratory analysis, we assessed the relationship between TAPSE/PASP and clinical outcomes. At a median follow-up of 865 days (517–1,461), 133 patients experienced the composite of death, LVAD implantation, or transplantation. Moreover, in the Kaplan–Meier analysis on the cohort, we observed that survival decreased as the TAPSE/PASP tertiles increased (Figure 3). In the multivariable Cox regression analysis, the confounders identified by the DAG were included as covariates, while peak VO₂ was not incorporated into the model as it was considered a mediator. In the multivariable Cox model, a 0.1-unit increase in TAPSE/PASP was associated with a significantly lower risk of the composite endpoint (HR: 0.78; 95% CI: 0.68–0.90; *p* < 0.001) (Table 6; Supplementary Figure S4), corresponding to an approximately 22% relative risk reduction per 0.1-unit increase. In a sensitivity analysis restricted to all-cause mortality, with LVAD implantation and heart transplantation treated as censoring events, TAPSE/PASP remained independently associated with risk. A 0.1-unit increase in TAPSE/PASP was associated with a lower mortality risk (HR: 0.68; 95% CI: 0.55–0.83; *p* < 0.001) (Supplementary Table S5).

**Table 6:** Cox regression analysis to assess the prognostic impact of the TAPSE/PASP ratio on clinical outcomes.

Variable	HR (Univariable)	95% CI	*p*-Value	HR (Multivariable)	95% CI	*p*-Value
Gender (female vs. male)	1.40	0.72–2.72	0.316	1.27	0.60–2.70	0.532
Ischemic etiology (yes vs. no)	0.83	0.54–1.27	0.390	1.04	0.61–1.74	0.896
Age (per year)	1.00	0.98–1.02	0.898	0.99	0.97–1.02	0.541
Left ventricular ejection fraction (% per unit)	0.91	0.86–0.97	0.003	0.98	0.91–1.05	0.504
TAPSE/PASP (per 0.1-unit increase)	0.78	0.69–0.88	<0.001	0.82	0.71–0.95	0.008
Cardiac index (per L/min/m^2^)	0.57	0.35–0.95	0.030	1.02	0.53–1.93	0.962
Pulmonary vascular resistance (per WU)	1.07	1.01–1.13	0.028	1.00	0.93–1.08	0.962
Hemoglobin (per g/dL)	0.85	0.76–0.95	0.003	0.93	0.81–1.08	0.339
GFR (per mL/min/1.73 m^2^)	0.99	0.99–1.00	0.194	1.00	0.99–1.01	0.717
INR	1.56	1.11–2.19	0.010	1.37	0.89–2.12	0.156
Albumin (per g/L)	0.94	0.91–0.97	<0.001	0.97	0.92–1.02	0.240
RAP (mmHg)	1.04	1.01–1.07	0.007	1.01	0.98–1.05	0.455
BMI (kg/m^2^)	0.95	0.91–0.99	0.022	0.95	0.90–1.00	0.038

BMI, body mass index; CI, confidence interval; INR, international normalized ratio; PASP, pulmonary artery systolic pressure; PVR, pulmonary vascular resistance; RAP, right atrial pressure; HR, hazard ratio; TAPSE, tricuspid annular plane systolic excursion.

## Discussion

This study is, to our knowledge, among the first to examine the potential association between the TAPSE/PASP ratio and exercise capacity in heart transplant candidates with advanced HF. In addition, we explored the relationship between TAPSE/PASP and long-term outcomes in secondary analyses. Unlike prior work in broader HF populations, our analysis focused on a highly selected transplant candidate cohort and employed a DAG approach to guide confounder selection and minimize potential bias. This methodological strategy enabled structured identification of minimally sufficient adjustment sets.

Our findings indicate that TAPSE/PASP may reflect more than a static measure of right ventricular (RV) function. The observed association with peak oxygen consumption (VO_₂_) suggests that RV–pulmonary artery (PA) coupling may be related to functional capacity, which in turn is known to be linked to long-term prognosis. Notably, a lower TAPSE/PASP ratio was also associated with a higher risk of adverse clinical outcomes, potentially reflecting its relationship with exercise capacity. Although TAPSE/PASP demonstrated an association with the composite clinical endpoint, these prognostic results should be interpreted as complementary to—rather than central to—the primary aim of the study. The analysis was not designed to establish causal relationships for clinical outcomes, and the survival findings are therefore hypothesis-generating.

Exercise capacity is widely recognized as a strong prognostic indicator in advanced HF and plays an important role in transplant evaluation (14). Previous studies have reported that reduced peak VO₂ is associated with increased mortality and may influence heart transplant listing decisions (3, 20). Our results add to this body of evidence by suggesting that TAPSE/PASP, a readily obtainable echocardiographic parameter, is associated with functional performance in advanced HF. Thus, TAPSE/PASP may complement established functional markers in pre-transplant assessment.

The observed reduction in VE/VCO₂ slope across TAPSE/PASP tertiles may also reflect improved RV–PA coupling. During exercise, effective coupling permits adequate augmentation of pulmonary perfusion, thereby reducing physiological dead space ventilation and decreasing ventilatory drive. Conversely, impaired coupling results in elevated pulmonary pressures, ventilation–perfusion mismatch, and excessive chemoreceptor activation, all of which contribute to a steeper VE/VCO_₂_ slope. This mechanism supports the interdependence between right-sided hemodynamics and ventilatory efficiency in advanced HF.

RV function is determined by preload, afterload, myocardial contractility, and pericardial constraint. TAPSE/PASP has been proposed as an index of RV–PA coupling, integrating RV systolic function with afterload. Prior studies have linked low TAPSE/PASP values with increased mortality in HF populations (21, 22), although few have examined its relationship to exercise performance. Our results suggest that impaired RV–PA coupling may be associated with exercise intolerance, possibly by limiting the ability to augment stroke volume during exertion, thereby restricting cardiac output and oxygen delivery. These observations align with the hypothesis proposed by Teramoto et al. (7), who reported that elevated pulmonary pressures and reduced RV function were associated with lower peak VO_₂_.

Despite its clinical utility, several methodological limitations of the TAPSE/PASP ratio should be acknowledged. TAPSE reflects only longitudinal shortening of the right ventricle and therefore may underestimate global RV systolic performance, particularly in patients with prior cardiac surgery, annular tethering, or regional dysfunction. Its accuracy is also reduced in the presence of significant tricuspid regurgitation, where annular motion may not reliably represent true contractility. Likewise, Doppler-derived PASP estimation is dependent on the quality and direction of the TR jet and may be inaccurate when the regurgitant jet is eccentric or poorly visualized. Consequently, while the TAPSE/PASP ratio remains a practical, reproducible, and widely studied surrogate of RV–PA coupling, it should be interpreted within the context of these physiological and technical constraints (19).

Beyond these methodological strengths, TAPSE/PASP also offers practical advantages. Its non-invasive, reproducible measurement by transthoracic echocardiography allows for repeated assessments over time, facilitating monitoring of disease progression. If validated in larger, multicenter studies, incorporating TAPSE/PASP into transplant evaluation protocols could help refine candidate selection, improve risk stratification, and inform the timing of interventions such as LVAD implantation.

It is important to note that the proportion of patients who ultimately underwent heart transplantation in our cohort was relatively low. This pattern reflects national and center-specific factors rather than disease severity. In Turkey, donor organ availability remains limited, resulting in prolonged waiting times and reducing the number of patients who can proceed to transplantation. In addition, LVADs are frequently used as destination therapy rather than exclusively as a bridge to transplant, further decreasing transplant rates. Moreover, a proportion of patients died or experienced clinical deterioration before a suitable donor organ became available. These contextual factors explain the lower-than-expected transplantation frequency in this advanced HF population.

### Study limitations

Several limitations should be considered when interpreting these results. First, TAPSE/PASP was measured at rest, whereas peak VO_₂_ was obtained during maximal exercise. This temporal mismatch may not fully capture dynamic changes in RV–PA coupling under physiological stress. The absence of exercise echocardiography or stress hemodynamic data limits direct assessment of coupling during exertion.

Second, this was a single-center observational study without a formal sample size calculation. Complete-case analysis may have introduced selection bias. The cohort was restricted to patients with LVEF ≤25% and included a relatively small proportion of women (14%), limiting generalizability to broader HF populations. Although advanced HF is not defined solely by LVEF and some patients with LVEF >25% may still fulfill guideline-based criteria for advanced HF (e.g., restrictive physiology, infiltrative disease, or predominant RV failure), these patients were excluded to maintain a physiologically homogeneous cohort with severe systolic dysfunction and to avoid mixing heterogeneous HF phenotypes. This methodological decision enhances internal consistency but limits generalizability to patients with moderately reduced EF who may nonetheless be considered for transplantation.

Third, TAPSE/PASP was analyzed using tertiles for statistical purposes, but standardized cut-off values for clinical decision-making have not been established. Inter- and intra-observer reproducibility was not assessed, and measurement accuracy may be affected in conditions such as prior cardiac surgery or severe tricuspid regurgitation.

Fourth, the exclusive use of treadmill-based CPET may have excluded patients unable to perform treadmill exercise, potentially affecting representativeness.

Fifth, INTERMACS profiles were not routinely collected during the study period and therefore could not be reported. This limits granular phenotyping of advanced HF status. In addition, despite the young age of the cohort, the proportion of patients who ultimately underwent transplantation was low. This pattern reflects national limitations in donor organ availability and reliance on LVAD as bridge-to-transplant or destination therapy, which may affect external applicability to regions with different allocation practices.

Sixth, RV fractional area change and RV free-wall longitudinal strain were not systematically acquired during routine imaging in the study period; therefore, alternative measures of RV systolic performance could not be evaluated in supplementary analyses. This limits the ability to compare TAPSE/PASP with other non-invasive RV–PA coupling indices.

Finally, although a DAG-based approach was used to reduce bias, unmeasured confounding cannot be entirely excluded. Other factors—such as chronotropic incompetence, peripheral muscle dysfunction, or ventilatory limitations—may partly explain the observed associations. The composite endpoint included transplantation, which in some cases may reflect therapeutic success rather than an adverse outcome, potentially influencing interpretation of survival analyses.

## Conclusion

In heart transplant candidates with advanced HF, the TAPSE/PASP ratio was associated with peak VO_₂_, exercise capacity, and long-term outcomes. These findings suggest that TAPSE/PASP may provide prognostic information beyond its role as a marker of RV systolic function. Owing to its simplicity, non-invasive nature, and reproducibility, TAPSE/PASP may support transplant evaluation and risk stratification. Further multicenter studies, particularly those incorporating exercise or stress-derived RV–PA coupling measurements, are warranted to confirm these associations and to determine clinically relevant cut-off values.

## Data Availability

The raw data supporting the conclusions of this article will be made available by the authors, without undue reservation.

## Supplementary material

Supplementary Figure S1 STROBE patient flow diagram. Flow diagram illustrating the number of patients screened at the advanced heart failure clinic (n = 439), the reasons for exclusion (n = 55; LVEF >25%, severe lung disease, contraindications to CPET or right heart catheterization, or peak RER <1.05), and the final cohort included in the analysis (n = 384) who underwent comprehensive echocardiography, CPET, and right heart catheterization.

upplementary Figure S2 Directed acyclic graph (DAG) illustrating the assumed relationships linking the TAPSE/PASP ratio to peak VO₂. RV-PA coupling (TAPSE/PASP) is modeled as the exposure and peak VO₂ as the outcome. Pink nodes indicate common ancestors of exposure and outcome, blue nodes indicate ancestors of the outcome, and green arrows represent assumed causal paths. Variables selected for adjustment in multivariable analysis included age, gender, BMI, ischemic etiology, pulmonary vascular resistance (PVR), cardiac index (CI), and hemoglobin. Peak VO₂ was not adjusted for in the Cox model as it was considered a mediator.

Supplementary Figure S3 Correlation matrix of echocardiographic and invasive PASP and TAPSE/PASP. Pulmonary artery systolic pressure (PASP) measured by echocardiography showed a strong positive correlation with invasively measured PASP (r = 0.615, p <0.001). Echocardiographic TAPSE/PASP demonstrated excellent agreement with invasive TAPSE/PASP (r = 0.766, p <0.001). Scatterplots display pairwise relationships with fitted regression lines and 95% confidence intervals. Negative correlations reflect expected inverse coupling patterns between TAPSE/PASP and PASP.

Supplementary Figure S4 Adjusted hazard ratio across the TAPSE/PASP continuum. Spline analysis shows an inverse relationship between TAPSE/PASP and the risk of death, LVAD implantation, or transplantation, with risk sharply increasing at lower TAPSE/PASP values.
