# Supplementary figures and images for: Association between the TAPSE/PASP ratio and exercise capacity in heart transplant candidates with advanced heart failure
Source: Front Cardiovasc Med. 2026 Jan 21;12:1686578. doi: 10.3389/fcvm.2025.1686578 (PMC12869431; doi:10.3389/fcvm.2025.1686578)

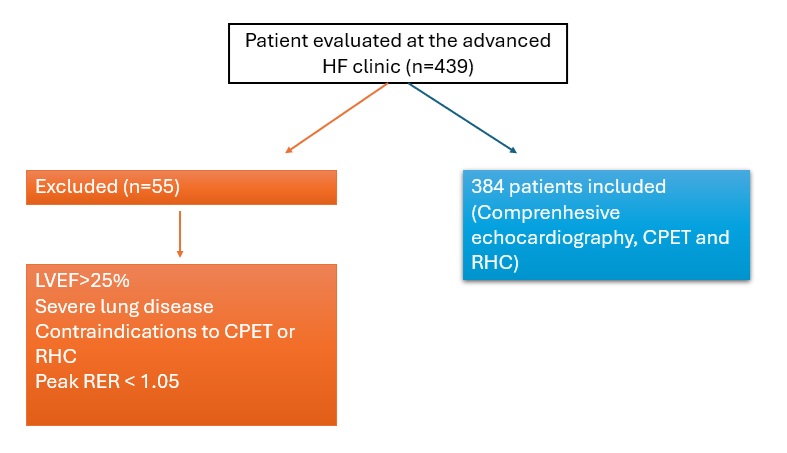

Supplement: Supplementary Figure S1 — STROBE patient flow diagram. Flow diagram illustrating the number of patients screened at the advanced heart failure clinic (n = 439), the reasons for exclusion (n = 55; LVEF >25%, severe lung disease, contraindications to CPET or right heart catheterization, or peak RER <1.05), and the final cohort included in the analysis (n = 384) who underwent comprehensive echocardiography, CPET, and right heart catheterization. [file Image1.jpeg]

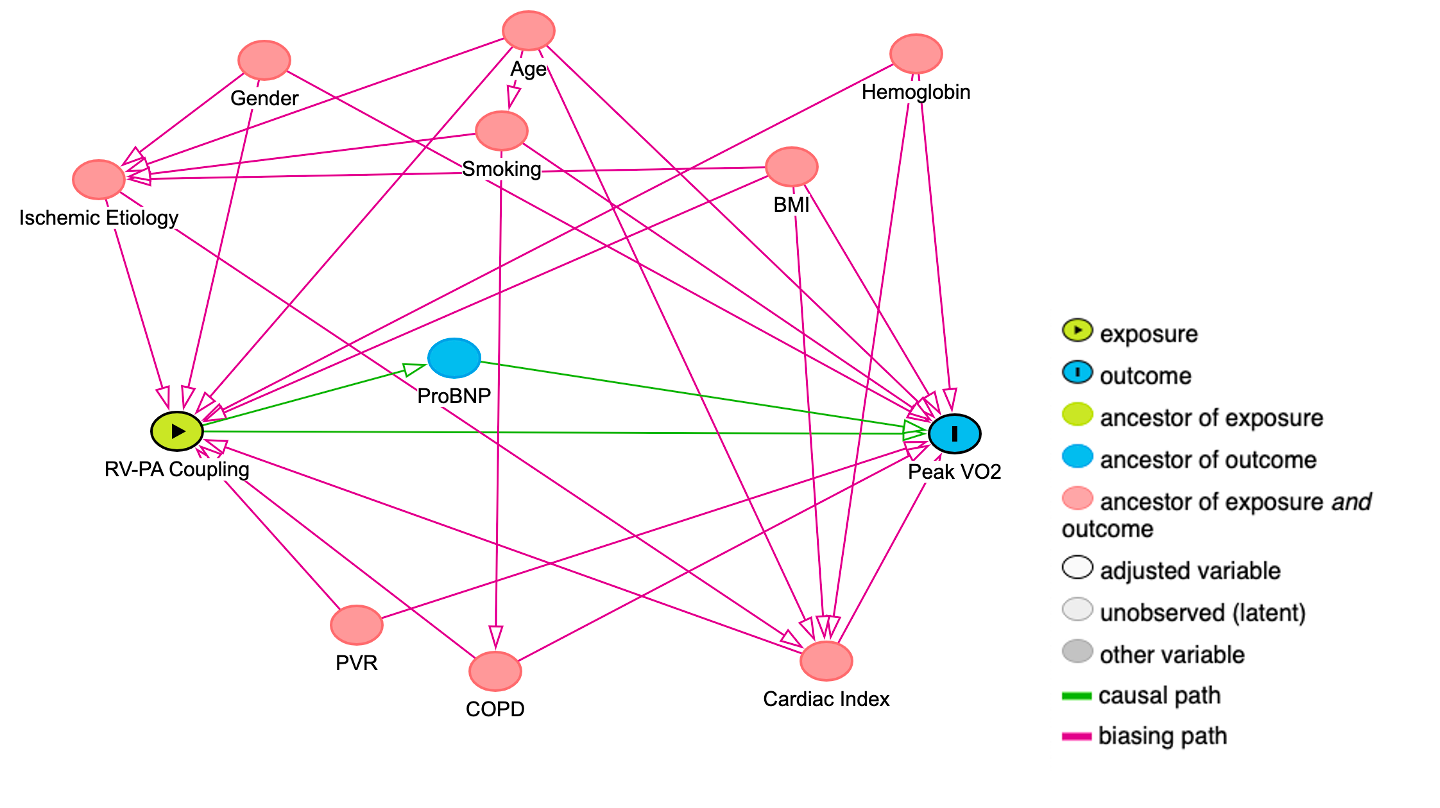

Supplement: upplementary Figure S2 — Directed acyclic graph (DAG) illustrating the assumed relationships linking the TAPSE/PASP ratio to peak VO₂. RV-PA coupling (TAPSE/PASP) is modeled as the exposure and peak VO₂ as the outcome. Pink nodes indicate common ancestors of exposure and outcome, blue nodes indicate ancestors of the outcome, and green arrows represent assumed causal paths. Variables selected for adjustment in multivariable analysis included age, gender, BMI, ischemic etiology, pulmonary vascular resistance (PVR), cardiac index (CI), and hemoglobin. Peak VO₂ was not adjusted for in the Cox model as it was considered a mediator. [file Image2.png]

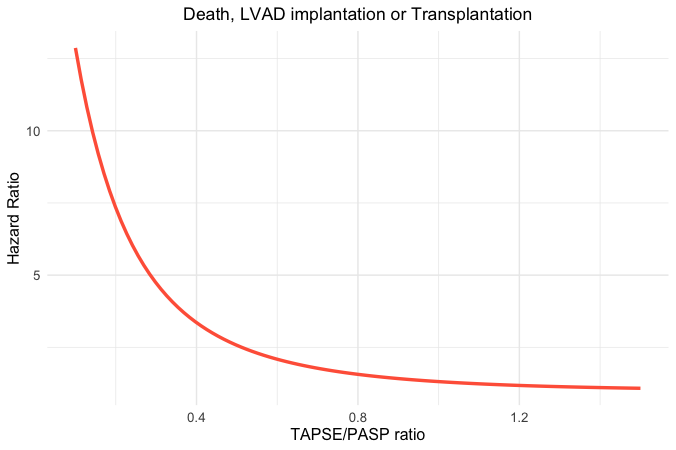

Supplement: Supplementary Figure S3 — Correlation matrix of echocardiographic and invasive PASP and TAPSE/PASP. Pulmonary artery systolic pressure (PASP) measured by echocardiography showed a strong positive correlation with invasively measured PASP (r = 0.615, p <0.001). Echocardiographic TAPSE/PASP demonstrated excellent agreement with invasive TAPSE/PASP (r = 0.766, p <0.001). Scatterplots display pairwise relationships with fitted regression lines and 95% confidence intervals. Negative correlations reflect expected inverse coupling patterns between TAPSE/PASP and PASP. [file Image3.tiff]

Correlation matrix of echocardiographic and invasive PASP and TAPSE/PASP

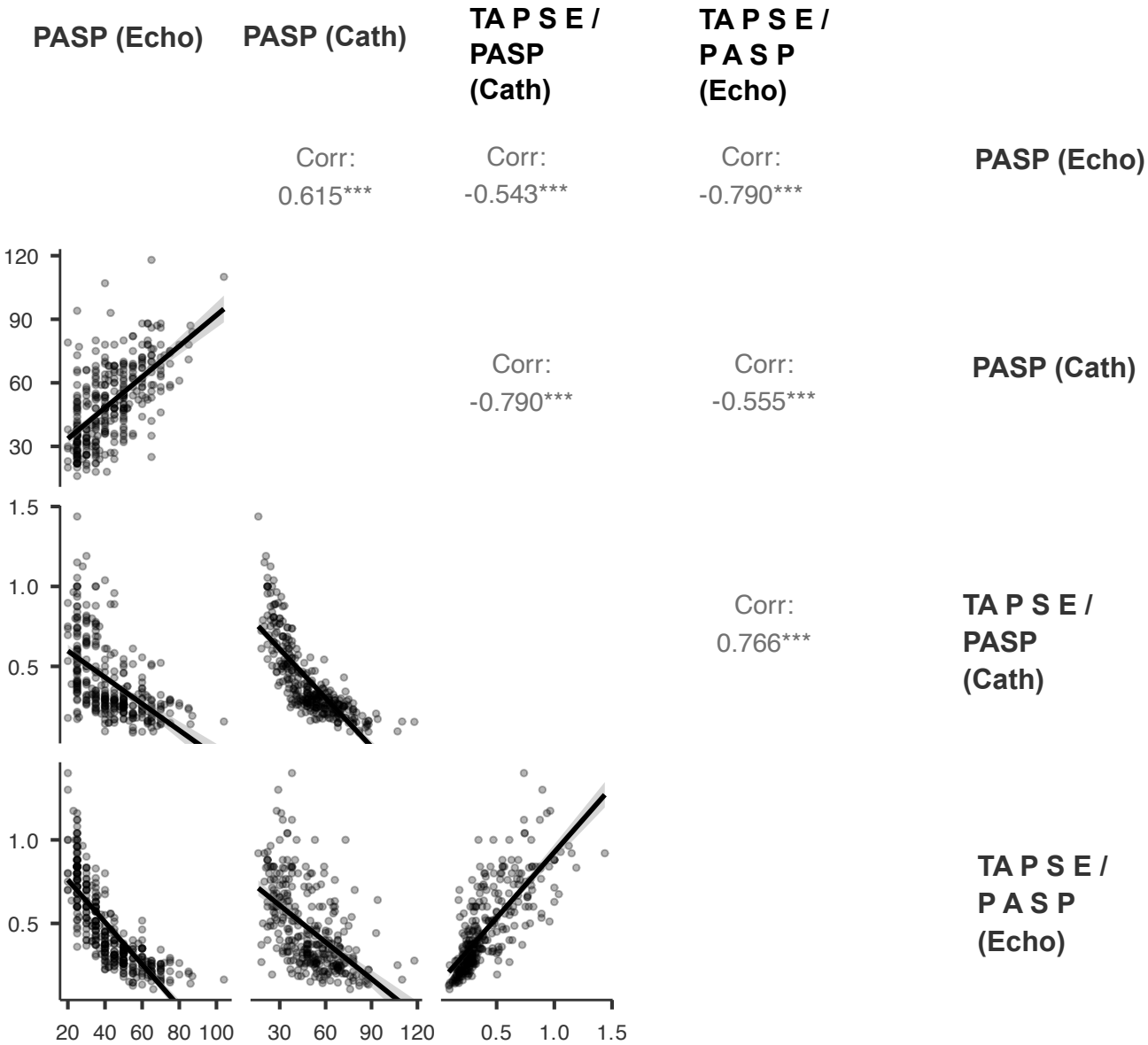

Supplement: Supplementary Figure S4 — Adjusted hazard ratio across the TAPSE/PASP continuum. Spline analysis shows an inverse relationship between TAPSE/PASP and the risk of death, LVAD implantation, or transplantation, with risk sharply increasing at lower TAPSE/PASP values. [file Image4.pdf]
