# Supplementary material for: Association between the TAPSE/PASP ratio and exercise capacity in heart transplant candidates with advanced heart failure
Source: Front Cardiovasc Med. 2026 Jan 21;12:1686578. doi: 10.3389/fcvm.2025.1686578 (PMC12869431; doi:10.3389/fcvm.2025.1686578)
Supplement: Supplementary file 5 [file Datasheet1.pdf]

Supplementary Table -1: Pharmacological and Device Therapy in the Study Cohort (n = 384)

Variable	n (%)
**Pharmacological Therapy**	
ACEI/ARB	114 (29.7)
ARNI	97 (25.3)
Beta-blocker	357 (93.0)
Mineralocorticoid receptor antagonist (MRA)	311 (81.0)
SGLT2 inhibitor	180 (46.9)
Loop diuretic	365 (95.1)
Anticoagulation	142 (37.0)
Statin	162 (42.2)
**Device Therapy**	
ICD	189 (49.2)
CRT	40 (10.4)

Abbreviations: ACEI: Angiotensin-converting enzyme inhibitor; ARB: Angiotensin receptor blocker; ARNI: Angiotensin receptor–neprilysin inhibitor; MRA: Mineralocorticoid receptor antagonist; SGLT2: Sodium–glucose cotransporter-2; ICD: Implantable cardioverter-defibrillator; CRT: Cardiac resynchronization therapy.

Supplementary Table-2: Multivariable Linear Regression Analysis for VE/VCO_2 Slope

Predictor	Estimate (β)	Standard Error	t-value	p-value
Intercept	86.589	11.246	7.700	<0.001
Age (per year)	0.158	0.097	1.630	0.104
Gender (female vs male)	0.378	3.042	0.124	0.901
BMI (kg/m²)	−0.161	0.200	−0.804	0.422
Ischemic etiology (yes vs no)	−4.406	2.226	−1.980	0.049
PVR (per WU)	0.447	0.447	1.002	0.317
Cardiac index (per L/min/m²)	−5.152	2.369	−2.175	0.030
Hemoglobin (per g/dL)	−1.705	0.534	−3.193	0.002
TAPSE/PASP (per unit)	**−21.189**	**4.957**	**−4.275**	**<0.001**

Abbreviations: BMI: Body mass index; PVR: Pulmonary vascular resistance; CI: Cardiac index; TAPSE: Tricuspid annular plane systolic excursion; PASP: Pulmonary artery systolic pressure; WU: Wood units.

Supplementary Table-3: Pearson Correlation Matrix Among TAPSE/PASP, VE/VCO_2 Slope, and Peak VO_2

Variable Pair	Pearson's r	df	p-value
TAPSE/PASP – VE/VCO_2 slope	–0.395	374	<0.001
TAPSE/PASP – peak VO_2	0.524	381	<0.001
VE/VCO_2 slope – peak VO_2	–0.599	400	<0.001

Abbreviations: TAPSE: Tricuspid annular plane systolic excursion; PASP: Pulmonary artery systolic pressure; VO_2: Oxygen consumption; VE/VCO_2 slope: Ventilatory efficiency slope; df: Degrees of freedom.

Supplementary Table-4: Multivariable Linear Regression Analysis for % Predicted Peak VO_2

Predictor	Estimate (β)	SE	t-value	p-value
Intercept	53.421	5.835	9.156	<0.001
Age (per year)	–0.308	0.051	–6.029	<0.001
Gender (female vs male)	5.577	1.590	3.507	<0.001
BMI (kg/m²)	–0.610	0.105	–5.795	<0.001
Ischemic etiology (yes vs no)	0.917	1.166	0.786	0.432
PVR (per WU)	0.057	0.233	0.244	0.807
Cardiac index (per L/min/m²)	1.232	1.250	0.986	0.325
Hemoglobin (per g/dL)	0.018	0.279	0.063	0.950
TAPSE/PASP (per unit)	**5.375**	**2.603**	**2.065**	**0.040**

Abbreviations: BMI: Body mass index; CI: Cardiac index; PVR: Pulmonary vascular resistance; VO_2: Oxygen consumption; % predicted VO_2: Percent-predicted peak oxygen consumption; SE: Standard error; TAPSE: Tricuspid annular plane systolic excursion; PASP: Pulmonary artery systolic pressure.

Supplementary Table-5: Multivariable Cox Regression for All-Cause Mortality (Sensitivity Analysis)

Variable	HR (Multivariable)	95% CI	p-value
Gender (female vs male)	1.27	0.60–2.70	0.532
Ischemic etiology (yes vs no)	1.04	0.61–1.74	0.896
Age (per year)	0.99	0.97–1.02	0.541
Left ventricular ejection fraction (% per unit)	0.98	0.91–1.05	0.504
TAPSE/PASP (per 0.1-unit increase)	**0.68**	**0.55–0.83**	**<0.001**
Cardiac index (per L/min/m²)	1.02	0.53–1.93	0.962
Pulmonary vascular resistance (per WU)	1.00	0.93–1.08	0.962
Hemoglobin (per g/dL)	0.93	0.81–1.08	0.339
GFR (per mL/min/1.73m²)	1.00	0.99–1.01	0.717
INR	1.37	0.89–2.12	0.156
Albumin (per g/L)	0.97	0.92–1.02	0.240
RAP (mmHg)	1.01	0.98–1.05	0.455
BMI (kg/m²)	0.95	0.90–1.00	0.038

Abbreviations: BMI: Body mass index, CI: Confidence interval, INR: International Normalized Ratio, PASP: Pulmonary artery systolic pressure, PVR: Pulmonary vascular resistance, RAP: Right atrial pressure, HR: Hazard ratio, TAPSE: Tricuspid annular plane systolic excursion.
